# Supplementary material for: Identification of the Actinomycin D Biosynthetic Pathway from Marine-Derived Streptomyces costaricanus SCSIO ZS0073
Source: Mar Drugs. 2019 Apr 23;17(4):240. doi: 10.3390/md17040240 (PMC6521150; doi:10.3390/md17040240)
Supplement: Supplementary file 1 [file marinedrugs-17-00240-s001.pdf]

Supplementary Materials

Identification of the Actinomycin D Biosynthetic Pathway from Marine-derived *Streptomyces costaricanus* SCSIO ZS0073

Mengchan Liu[1,2,3], Yanxi Jia[1,2,3], Yunchang Xie[1], Chunyan Zhang[1,2], Juying Ma[1,2], Changli Sun[1], Jianhua Ju[1,2*]

[1] CAS Key Laboratory of Tropical Marine Bio-resources and Ecology, Guangdong Key Laboratory of Marine Materia Medica, South China Sea Institute of Oceanology, Chinese Academy of Sciences, 164 West Xingang Road, Guangzhou 510301, China; 18696164503@163.com (M.L.) jiayanxi0928@163.com (Y.J.); xieyunchang@scsio.an.cn (Y.X.); zhchuny@foxmail.com (C.Z.); majunying@scsio.ac.cn (J.M.); lingluboxi@163.com (C.S.)

[2] College of Oceanography, University of Chinese Academy of Sciences, Beijing 100049, China.

[3] Contributed equally

* Correspondence: jju@scsio.ac.cn; Tel./Fax: +86-20-8902-3028

**Item Name**	**Contents/Description**	**Page**
**Table S1**	Structural Information summary for the actinomycins	S3-S4
**Table S2**	Summary of strains and plasmids used in this study.	S5-S7
**Table S3**	Summary of primers used in this study.	S8-S12
**Figure S1**	Disruption of *orf(-3)* in WT *S. costaricanus* SCSIO ZS0073.	S13
**Figure S2**	Disruption of *orf(-2)* in WT *S. costaricanus* SCSIO ZS0073.	S13
**Figure S3**	Disruption of *orf(-1)* in WT *S. costaricanus* SCSIO ZS0073.	S14
**Figure S4**	Disruption of *orf(+1)* in WT *S. costaricanus* SCSIO ZS0073.	S14
**Figure S5**	Disruption of *orf(+2)* in WT *S. costaricanus* SCSIO ZS0073.	S15
**Figure S6**	Disruption of *orf(+3)* in WT *S. costaricanus* SCSIO ZS0073.	S15
**Figure S7**	HPLC analyses of fermentation extracts (**Fig. S2**-**S7** mutants).	S16
**Figure S8**	Disruption of *acnA* in WT *S. costaricanus* SCSIO ZS0073.	S16
**Figure S9**	Disruption of *acnB* in WT *S. costaricanus* SCSIO ZS0073.	S17
**Figure S10**	Disruption of *ancU1* in WT *S. costaricanus* SCSIO ZS0073.	S17
**Figure S11**	Disruption of *acnU2* in WT *S. costaricanus* SCSIO ZS0073.	S18
**Figure S12**	Disruption of *acnC* in WT *S. costaricanus* SCSIO ZS0073.	S18
**Figure S13**	Disruption of *acnU3* in WT *S. costaricanus* SCSIO ZS0073.	S19
**Figure S14**	Disruption of *acnU4* in WT *S. costaricanus* SCSIO ZS0073.	S19
**Figure S15**	Disruption of *acnD–E* in WT *S. costaricanus* SCSIO ZS0073.	S20
**Figure S16**	Disruption of *acnN1* in WT *S. costaricanus* SCSIO ZS0073.	S20
**Figure S17**	Disruption of *acnN3* in WT *S. costaricanus* SCSIO ZS0073.	S21
**Figure S18**	Disruption of *acnG–P* in WT *S. costaricanus* SCSIO ZS0073.	S21
**Figure S19**	Disruption of *acnR* in WT *S. costaricanus* SCSIO ZS0073.	S22
**Figure S20**	Disruption of *acnQ* in WT *S. costaricanus* SCSIO ZS0073.	S22
**Figure S21**	Disruption of *acnT1* in WT *S. costaricanus* SCSIO ZS0073.	S23
**Figure S22**	Disruption of *acnT2* in WT *S. costaricanus* SCSIO ZS0073.	S23
**Figure S23**	Disruption of *acnT3* in WT *S. costaricanus* SCSIO ZS0073.	S24
**Figure S24**	Disruption of *acnW* in WT *S. costaricanus* SCSIO ZS0073.	S24
**Figure S25**	Disruption of *acnF* in WT *S. costaricanus* SCSIO ZS0073.	S25
**Figure S26**	Disruption of *acnU4* in WT *S. costaricanus* SCSIO ZS0073.	S25
**Figure S27**	Disruption of *acnP* in WT *S. costaricanus* SCSIO ZS0073.	S26
**Figure S28**	Disruption of *PHS* in WT *S. costaricanus* SCSIO ZS0073.	S26
**Figure S29**	HPLC-ESI-MS chromatogram of the fermentation extract of Δ*acnF*	S27

Table S1: Summary of structural information for the actinomycins

Name	*α*-ring (R_1)	*β*-ring (R_2)	chromophore	Type
Actinomycin D [a]	Thr-D-Val-Pro-Sar-MeVal	Thr-D-Val-Pro-Sar-MeVal	Actinocin (i)	Actinomycin D
Actinomycin D_0 [a]	Thr-D-Val-Pro-Sar-MeVal	Thr-D-Val-Pro-Gly-MeVal	Actinocin (i)	*N*-demethyl actinomycins
N,N′-Didemethyl-actinomycin D [a]	Thr-D-Val-Pro-Gly-MeVal	Thr-D-Val-Pro-Gly-MeVal	Actinocin (i)	
Actinomycin C_2 [a]	Thr-D-Val-Pro-Sar-MeVal	Thr-D-*a*Ile-Pro-Sar-MeVal	Actinocin (i)	C-type Actinomycin
Actinomycin C_{2a} [a]	Thr-D-*a*Ile-Pro-Sar-MeVal	Thr-D-Val-Pro-Sar-MeVal	Actinocin (i)	
Actinomycin C_3 [a]	Thr-D-*a*Ile-Pro-Sar-MeVal	Thr-D-*a*Ile-Pro-Sar-MeVal	Actinocin (i)	
Actinomycin F_8 [a]	Thr-D-Val-Sar-Sar-MeVal	Thr-D-Val-Sar-Sar-MeVal	Actinocin (i)	F-type Actinomycin
Actinomycin F_9 [a]	Thr-D-Val-Sar-Sar-MeVal<br>Thr-D-Val-Pro-Sar-MeVal	Thr-D-Val-Pro-Sar-MeVal<br>Thr-D-Val-Sar-Sar-MeVal	Actinocin (i)	
Actinomycin Xoα [a]	Thr-D-Val-Sar-Sar-MeVal	Thr-D-Val-Hyp-Sar-MeVal	Actinocin (i)	X-type Actinomycin
Actinomycin Xoβ [a]	Thr-D-Val-Pro-Sar-MeVal	Thr-D-Val-Hyp-Sar-MeVal	Actinocin (i)	
Actinomycin Xoδ [a]	Thr-D-Val-Pro-Sar-MeVal	Thr-D-Val-*a*Hyp-Sar-MeVal	Actinocin (i)	
Actinomycin X_{1a} [a]	Thr-D-Val-Sar-Sar-MeVal	Thr-D-Val-OPro-Sar-MeVal	Actinocin (i)	
Actinomycin X_2 [a]	Thr-D-Val-Pro-Sar-MeVal	Thr-D-Val-OPro-Sar-MeVal	Actinocin (i)	
Actinomycin Z_1 [a]	Thr-D-Val-HMPro-Sar-MeVal	HThr-D-Val-MOPro-Sar-MeAla	Actinocin (i)	Z-type Actinomycin
Actinomycin Z_2 [a]	Thr-D-Val-HMPro-Sar-MeVal	Thr-D-Val-MOPro-Sar-MeAla	Actinocin (i)	
Actinomycin Z_3 [a]	Thr-D-Val-HMPro-Sar-MeVal	ClThr-D-Val-MOPro-Sar-MeAla	Actinocin (i)	
Actinomycin Z_4 [a]	Thr-D-Val-MPro-Sar-MeVal	Thr-D-Val-MOPro-Sar-MeAla	Actinocin (i)	
Actinomycin Z_5 [a]	Thr-D-Val-MPro-Sar-MeVal	ClThr-D-Val-MOPro-Sar-MeAla	Actinocin (i)	
Actinomycin Z_6 [b]	Thr-D-Val-HMPro-Sar-MeVal	HThr-D-Val-MOPro-Sar-MeAla	Actinocin (i)	
Actinomycin ZP [a]	Thr-D-Val-MPro-Sar-MeVal	Thr-D-Val-MPro-Sar-MeVal	Actinocin (i)	
Actinomycin G1 [a]	Thr-D-Val-Pro-Sar-MeVal	HThr-D-Val-HMPro-Sar-MeAla	Actinocin (i)	G-type Actinomycin
Actinomycin G2 [a]	Thr-D-Val-HMPro-Sar-MeVal	ClThr-D-Val-Pro-Sar-MeAla	Actinocin (i)	
Actinomycin G3 [a]	Thr-D-Val-HMPro-Sar-MeVal	HThr-D-Val-Pro-Sar-MeAla	Actinocin (i)	
Actinomycin G4 [a]	Thr-D-Val-HMPro-Sar-MeVal	Thr-D-Val-Pro-Sar-MeAla	Actinocin (i)	
Actinomycin G5 [a]	Thr-D-Val-Pro-Sar-MeAla	cHThr-D-Val-Pro-Sar-MeAla	Actinocin (i)	
Actinomycin G6 [a]	Thr-D-Val-HMPro-Sar-MeVal	rHThr-D-Val-Pro-Sar-MeAla	Actinocin (i)	
Actinomycin Y1 [a]	Thr-D-Val-HMPro-Sar-MeVal	ClThr-D-Val-OPro-Sar-MeAla	Actinocin (i)	Y-type Actinomycin
Actinomycin Y2 [a]	Thr-D-Val-HMPro-Sar-MeVal	ClThr-D-Val-Hyp-Sar-MeAla	Actinocin (i)	
Actinomycin Y3 [a]	Thr-D-Val-HMPro-Sar-MeVal	rHThr-D-Val-OPro-Sar-MeAla	Actinocin (i)	
Actinomycin Y4 [a]	Thr-D-Val-HMPro-Sar-MeVal	rHThr-D-Val-Hyp-Sar-MeAla	Actinocin (i)	
Actinomycin Y5 [a]	Thr-D-Val-HMPro-Sar-MeVal	cThr-D-Val-OPro-Sar-MeAla	Actinocin (i)	
Actinomycin Y6 [a]	Thr-D-Val-HMPro-SarMeVal	crThr-D-Val-OPro-SarMeAla	Actinocin (i)	
Actinomycin Y7 [a]	Thr-D-Val-HMPro-SarMeVal	HThr-D-Val-OPro-SarMeAla	Actinocin (i)	
Actinomycin Y8 [a]	Thr-D-Val-HMPro-SarMeVal	Thr-D-Val-OPro-SarMeAla	Actinocin (i)	
Actinomycin Y9 [a]	Thr-D-Val-MPro-SarMeVal	Thr-D-Val-OPro-SarMeAla	Actinocin (i)	
methylated actinomycin D [c]	Thr-D-Val-Pro-Sar-MeVal	Thr-D-Val-Pro-Sar-MeVal	Modified chromophore (ii)	

Actinomycin D1 [d]	Thr-D-Val-Pro-Sar-MeVal	Thr-D-Val-Pro-Sar-MeVal	Modified chromophore (iv)	Actinomycin D analogues
Actinomycin D2 [d]	Thr-D-Val-Pro-Sar-MeVal	Thr-D-Val-Pro-Sar-MeVal	Modified chromophore (iv)	
Actinomycin D3 [d]	Thr-D- Val--Pro-Sar-MeVal	Thr-D-Val-Pro-Sar-MeVal	Modified chromophore (iii)	
Actinomycin D4 [d]	Thr-D-Ala--Pro-Sar-MeVal	Thr-D-Val-Pro-Sar-MeVal	chromophore (i)	
Neo-actinomycin A [e]	Thr-D-Val-Pro-Sar-MeVal	Thr-D-Val-Pro-Sar-MeVal	Modified chromophore (iv)	Neo-actinomycin
Neo-actinomycin B [e]	Thr-D-Val-Pro-Sar-MeVal	Thr-D-Val-Pro-Sar-MeVal	Modified chromophore (iv)	

References a-e:

a is taken from: Cai, W.L.; Wang, X.; Elshahawi, S.I.; Ponomareva, L.V.; Liu, X.; McErlean, M.R.; Cui, Z.; Arlinghaus, A.L.; Thorson, J.S.; Van Lanen, S.G. Antibacterial and cytotoxic actinomycins Y6–Y9 and Zp from *Streptomyces* sp. Strain Gö-GS12. *J. Nat. Prod.* **2016**, *79*, 2731-2739.

b is taken from: Dong, M.; Cao, P.; Ma, Y.T.; Luo, J.; Yan, Y.; Li, R.T.; Huang, S.X. A new actinomycin Z analogue with an additional oxygen bridge between chromophore and β-depsipentapeptide from *Streptomyces* sp. KIB-H714. *Nat. Prod. Res.* **2018**, *2*, 1-7.

c is taken from: Chen, Y.; Liu, J.; Yuan, B.; Cao, C.; Qin, S.; Cao, X.; Bian, G.; Wang, Z.; Jiang, J. Methylated actinomycin D, a novel actinomycin D analog induces apoptosis in HepG2 cells through Fas-and mitochondria-mediated pathways. *Mol. Carcinog.* **2013**, *52*, 983-996.

d is taken from: Jiao, W.H.; Yuan, W.; Li, Z.Y.; Li, J.; Li, L.; Sun, J.B; Gui, Y.H.; Wang, J.; Ye, B.P.; Lin, H.W. Anti-MRSA actinomycins D1-D4 from the marine sponge-associated *Streptomyces* sp. LHW52447. *Tetrahedron.* **2018**, *74*, 5914-5919.

e is taken from: Wang, Q.; Zhang, Y.; Wang, M.; Tan, Y.; Hu, X.; He, H.; Xiao, C.L.; You, X.; Wang Y.; Gan, M. Neo-actinomycins A and B, natural actinomycins bearing the 5 H-oxazolo [4, 5-b] phenoxazine chromophore, from the marine-derived *Streptomyces* sp. IMB094. *Sci. Rep.* **2017**, *7*, 3591.

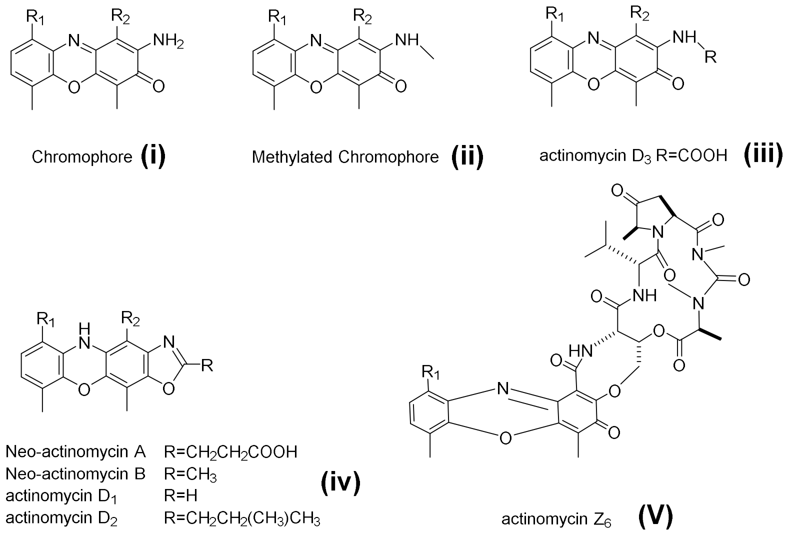

Modified chromophores characteristic of actinomycin analogues.

Table S2. Summary of strains and plasmids used in this study

Strains/ plasmids	Relevant phenotype	Source/[Ref]
S. SCSIO ZS0073	Wild-type (WT) producer of actinomycin	This work
Δ*orf(-3)*	*S. costaricanus* SCSIO ZS0073 with a 1039 bp of *orf(-3)* substituted by aac(3)IV+OriT	This work
Δ*orf(-2)*	*S. costaricanus* SCSIO ZS0073 with a 793 bp of *orf(-2)* substituted by aac(3)IV+OriT	This work
Δ*orf(-1)*	*S. costaricanus* SCSIO ZS0073 with a 2000 bp of *orf(-1)* substituted by aac(3)IV+OriT	This work
Δ*acnW*	*S. costaricanus* SCSIO ZS0073 with a 539 bp of Δ*acnW* ubstituted by aac(3)IV+OriT	This work
Δ*acnA*	*S. costaricanus* SCSIO ZS0073 with a 366 bp of Δ*acnA* substituted by aac(3)IV+OriT	This work
Δ*acnB*	*S. costaricanus* SCSIO ZS0073 with a 1213 bp of Δ*acnB* substituted by aac(3)IV+OriT	This work
Δ*acnU1*	*S. costaricanus* SCSIO ZS0073 with a 366 bp of Δ*acnU1* substitued by aac(3)IV+OriT	This work
Δ*acnU2*	*S. costaricanus* SCSIO ZS0073 with a 375 bp of Δ*acnU2* substituted by aac(3)IV+OriT	This work
Δ*acnC*	*S. costaricanus* SCSIO ZS0073 with a 742 bp of Δ*acnC* substituted by aac(3)IV+OriT	This work
Δ*acnU3*	*S. costaricanus* SCSIO ZS0073 with a 625 bp of Δ*acnU3* substituted by aac(3)IV+OriT	This work
Δ*acnU4*	*S. costaricanus* SCSIO ZS0073 with a 564 bp of Δ*acnU4* substituted by aac(3)IV+OriT	This work
Δ*acnD–E*	*S. costaricanus* SCSIO ZS0073 with a 456 bp of Δ*acnD-E* substituted by aac(3)IV+OriT	This work
Δ*acnN1*	*S. costaricanus* SCSIO ZS0073 with a 933 bp of Δ*acnN1* substituted by aac(3)IV+OriT	This work
Δ*acnN3*	*S. costaricanus* SCSIO ZS0073 with a 1021 bp of Δ*acnN3* substituted by aac(3)IV+OriT	This work
Δ*acnF*	*S. costaricanus* SCSIO ZS0073 with a 636 bp of Δ*acnF* substituted by aac(3)IV+OriT	This work
Δ*acnG–P*	*S. costaricanus* SCSIO ZS0073 with a 2300 bp ofΔ*acnG-P)* substituted by aac(3)IV+OriT	This work
Δ*acnR*	*S. costaricanus* SCSIO ZS0073 with a 893 bp of Δ*acnR* substituted by aac(3)IV+OriT	This work

Δ*acnQ*	*S. costaricanus* SCSIO ZS0073 with a 957 bp of Δ*acnQ* substituted by aac(3)IV+OriT	This work
Δ*acnT1*	*S. costaricanus* SCSIO ZS0073 with a 871 bp of Δ*acnT1* substituted by aac(3)IV+OriT	This work
Δ*acnT2*	*S. costaricanus* SCSIO ZS0073 with a 685 bp of Δ*acnT2* substituted by aac(3)IV+OriT	This work
Δ*orf(T3)*	*S. costaricanus* SCSIO ZS0073 with a 891 bp of Δ*orf(T3)* substituted by aac(3)IV+OriT	This work
Δ*orf(+1)*	*S. costaricanus* SCSIO ZS0073 with a 517 bp of Δ*orf(+1)* substituted by aac(3)IV+OriT	This work
Δ*orf(+2)*	*S. costaricanus* SCSIO ZS0073 with a 330 bp of Δ*orf(+2)* substituted by aac(3)IV+OriT	This work
Δ*orf(+3)*	*S. costaricanus* SCSIO ZS0073 with a 840 bp of Δ*orf(+3)* substituted by aac(3)IV+OriT	This work
Δ*phs*	*S. costaricanus* SCSIO ZS0073 with a 1694 bp of Δ*phs* substituted by aac(3)IV+OriT	This work
E.coli		
Bw25113	K-12 derivative: *araBAD, rhaBAD*	
ET12567	*dam, dcm, hsdM, hsdS, hsdR, catR , tetR*	
**Plasmids**		
pIJ773	P1-FRT-oriT-aac(3)IV-FRT-P2	
pIJ790	λ–RED (*gam bet exo*) CmlR *araCrep101ts*	
pUZ8002	*tra, neo*, RP4	This work
cosmid-9C2	A cosmid which contains partial actinomycin biosynthesis cluster	This work
cosmid-9A2	A cosmid which contains partial actinomycin biosynthesis cluster	This work
*p*Δ*orf(-3)*	A 1018 bp fragment in *orf(-3)* in cosmid 9A7 was substituted by the aac(IV)+OriT cassette using the PCR-targeting strategy	This work
*p*Δ*orf(-2)*	A 793 bp fragment in *orf(-2)* in cosmid 9A7 was substituted by the aac(IV)+OriT cassette using the PCR-targeting strategy	This work
*p*Δ*orf(-1)*	A 2000 bp fragment in *orf(-1)* in cosmid 9A7 was substituted by the aac(IV)+OriT cassette using the PCR-targeting strategy	This work
*p*Δ*acnW*	A 539 bp fragment in *acnW* in cosmid 9A7 was substituted by the aac(IV)+OriT cassette using the PCR-targeting strategy	This work
*p*Δ*acnA*	A 366 bp fragment in *acnA* in cosmid 9A7 was substituted by the aac(IV)+OriT cassette using the PCR-targeting strategy	This work
*p*Δ*acnB*	A 1229 bp fragment in *acnB* in cosmid 9A7 was substituted by the aac(IV)+OriT cassette using the PCR-targeting strategy	This work
*P*Δ*acnU1*	A 366 bp fragment in *acnU1* in cosmid 9A7 was substituted by the aac(IV)+OriT cassette using the PCR-targeting strategy	This work
*P*Δ*acnU2*	A 375 bp fragment in *acnU2* in cosmid 9A7 was substituted by the aac(IV)+OriT cassette using the PCR-targeting strategy	This work
*P*Δ*acnC*	A 742 bp fragment in *acnC* in cosmid 9A7 was substituted by the	This work

	aac(IV)+OriT cassette using the PCR-targeting strategy	
PΔacnU3	A 625 bp fragment in *acnU3* in cosmid 9A7 was substituted by the aac(IV)+OriT cassette using the PCR-targeting strategy	This work
PΔacnU4	A 564 bp fragment in *acnU4* in cosmid 9C2 was substituted by the aac(IV)+OriT cassette using the PCR-targeting strategy	This work
pΔacnD–E	A 458 bp fragment in *acnD-E* in cosmid 9C2 was substituted by the aac(IV)+OriT cassette using the PCR-targeting strategy	This work
pΔacnN1	A 933 bp fragment in *acnN1* in cosmid 9C2 was substituted by the aac(IV)+OriT cassette using the PCR-targeting strategy	This work
pΔacnN3	A 1044 bp fragment in *acnN3* in cosmid 9C2 was substituted by the aac(IV)+OriT cassette using the PCR-targeting strategy	This work
pΔacnF	A 636 bp fragment in *acnF* in cosmid 9C2 was substituted by the aac(IV)+OriT cassette using the PCR-targeting strategy	This work
pΔacnG–P	A 2300 bp fragment in *acnG-P* in cosmid 9C2 was substituted by the aac(IV)+OriT cassette using the PCR-targeting strategy	This work
pΔacnR	A 893 bp fragment in *acnR* in cosmid 9C2 was substituted by the aac(IV)+OriT cassette using the PCR-targeting strategy	This work
pΔacnQ	A 957 bp fragment in *acnQ* in cosmid 9C2 was substituted by the aac(IV)+OriT cassette using the PCR-targeting strategy	This work
pΔacnT1	A 871 bp fragment in *acnT1* in cosmid 9C2 was substituted by the aac(IV)+OriT cassette using the PCR-targeting strategy	This work
pΔacnT2	A 685 bp fragment in *acnT2* in cosmid 9C2 was substituted by the aac(IV)+OriT cassette using the PCR-targeting strategy	This work
pΔacnT3	A 891 bp fragment in *acnT3)* in cosmid 9A7 was substituted by the aac(IV)+OriT cassette using the PCR-targeting strategy	This work
pΔorf(+1)	A 517 bp fragment in *orf(+1)* in cosmid 9A7 was substituted by the aac(IV)+OriT cassette using the PCR-targeting strategy	This work
pΔorf(+2)	A 330 bp fragment in *orf(+2)* in cosmid 9A7 was substituted by the aac(IV)+OriT cassette using the PCR-targeting strategy	This work
pΔorf(+3)	A 840 bp fragment in *orf(+3)* in cosmid 9C2 was substituted by the aac(IV)+OriT cassette using the PCR-targeting strategy	This work
PHS	A 1694 bp fragment in *PHS* in cosmid 9C2 was substituted by the aac(IV)+OriT cassette using the PCR-targeting strategy	

Table S3 Summary of primers used in this study

Primer Name	Sequence (5'→3')	purpose
screen-73D-SF	TCTTGCCGGTGCCGGGCGGCC	For the screening of the genomic library
screen-73D-SF	TCCGGTGGGCAGGCGATCCGG	
screen-73ZA-F	GAGCAGCAGCGACTGCGCGCC	For the screening of the genomic library
screen-73ZA-R	TCAGCGCCGCACCCTCGGCAT	
screen-73DZB-F	CTGGCGCGTCCTCCTGCCGG	For the screening of the genomic library
screen-73DZBR	TCATTTCTGAATCCTCCACG	
screen-73DX-F	ACCGCGGCCCGGGAGCGCGC	For the screening of the genomic library
screen-73DX-R	ACGTTGGTCTTGGCCGAGCCG	
screen-73FS-F	TGACATCGCCGTCATCGGTCT	For the screening of the genomic library
screen-73FS-R	ACGTTGGTCTTGGCCGAGCCG	
screen-73FZ-F	AACTCCAGGGGCTCGGCGCCAT	For the screening of the genomic library
screen-73FZ-R	TGGTGAGGACCCCGGTGGCGTT	
screen-73FX-F	ACACCGTGCTGCGGCCGAAGG	For the screening of the genomic library
screen-73FX-R	TCAGAGAGACCCGAGCTCTTG	
orf(-3)-Del-PF	TGAGCCGGGCGCCCTGGGTGCTCTCGCGCAGGCACACCG attccggggatccgtcgacc	For disrupting *orf(-3)*
orf(-3)-Del-PR	GTGCAGACCCGACGCGATCACATGCAGGCGTACCAGTTC tgtaggctggagctgcttc	
orf(-3)-Ts-PF	TGCTCTCGCGCAGGCACACC	For verifying mutant Δ *orf(-3)*
orf(-3)-Ts-PR	CAGACCCGACGCGATCACAT	
orf(-2)-Del-PF	GGAGAAGTGGGAAGGGTGCGTATGCCGCGCATGCCGCTC attccggggatccgtcgacc	For disrupting *orf(-2)*
orf(-2)-Del-PR	CACTCCGCGCAACTGGCCGCCGCCGGGCAGGAGATCACC tgtaggctggagctgcttc	
orf(-2)-Ts-PF	TGACCGCCGTGCCAGTGGGT	For verifying mutant Δ *orf(-2)*
orf(-2)-Ts-PR	TCGGAGGTCTGCCGGGTCAC	
orf(-1)-Del-PF	GGAGAACAGGTCGTAGTCGTCGATGAGGACGAACAGCAG attccggggatccgtcgacc	For disrupting *orf(-1)*
orf(-1)-Del-PR	CTGGGCACCAAGTTCGAGCTGCGGCTGGGCGATTCGATG tgtaggctggagctgcttc	
orf (-1)-Ts-PF *orf(-1)-Ts-PR*	CTTGGCCTCGCCGAGGAACTTG GCGATGTGTTCCTGGTGGTC	For verifying mutant Δ *orf(-1)*
acnW-Del-PF	GGAGAAGTGGGAAGGGTGCGTATGCCGCGCATGCCGCTC attccggggatccgtcgacc	For disrupting *acnW*
acnW-Del-PR	CACTCCGCGCAACTGGCCGCCGCCGGGCAGGAGATCACC tgtaggctggagctgcttc	
acnW-Ts-PF	GGAGAAGTGGGAAGGGTGCG	For verifying mutant

acnW -Ts-PR	CACTCCGCGCAACTGGCCGC	Δ *acnW*
acnA-Del-PF	GTGGACGACTGGGACATGTGGGACAGGCGCGAGAGGACC attccggggatccgtcgacc	
acnA-Del-PR	TCAGAGCCAGGAGCTCGCCCGCTCCCCCGCCCGCCCCCG tgtaggctggagctgcttc	For disrupting *acnA*
acnA-Ts-PF	ACTGGGACATGTGGGACAGG	For verifying mutant Δ *acnA*
acnA-Ts-PR	AGAGCCAGGAGCTCGCCCGCT	
acnB-Del-PF	GGGCGCGCTGCTGCTGCGCATGTCCGGCACCCTGGACGC attccggggatccgtcgacc	
acnB-Del-PR	TCAGGGCCTGCGGGTCAGCCGGTGATGCAGATGTTCCAG tgtaggctggagctgcttc	For disrupting *acnB*
acnB-Ts-PF	GCACCGGCCCCCCTGCTGAT	For verifying mutant Δ*acnB*
acnB-Ts-PR	TGCGGGTCAGCCGGTGATGC	
acnU1-Del-PF	TCAGTGAGCGCCGAGCCCCCCGCCTCCGAAGGACCCGCT attccggggatccgtcgacc	
acnU1-Del-PR	ATGAACACCGCATCGATGCAGCTGGCCGCGACGAGCGGC tgtaggctggagctgcttc	For disrupting *acnU1*
acnU1-Ts-PF	CCGCCTCCGAAGGACCCGCT	For verifying mutant Δ*acnU1*
acnU1-Ts-PR	AACACCGCATCGATGCAGCTG	
acnU2-Del-PF	GTGACCCAGCAGGCTTCCCCGCAGCAGCTCTACCGGTTC attccggggatccgtcgacc	
acnU2-Del-PR	TCAGGCCAGCGTCTCCAGGAAGTCGACGCAGGCGCCGGC tgtaggctggagctgcttc	For disrupting *acnU2*
acnU2-Ts-PF	TGCGTCGACTTCCTGGAGAC	For verifying mutant Δ*acnU2*
acnU2-Ts-PR	TCTCCAGGAAGTCGACGCA	
acnC-Del-PF	GAGCAGCAGCGACTGCGCGCCGAACTGCGCGCCTACTTC attccggggatccgtcgacc	
acnC-Del-PR	CGCGGACGGCCGTCGTGCCGTGCGCCGCGAGGGTGACGC tgtaggctggagctgcttc	For disrupting *acnC*
acnC-Ts-PF	GAGCAGCAGCGACTGCGCGC	For verifying mutant Δ*acnC*
acnC-Ts-PR	CGCGGACGGCCGTCGTGCCG	
acnU3-Del-PF	CTAGCCGTAAGCCCGTTCGCTCTCCCGGTAGCGCTCCAG attccggggatccgtcgacc	
acnU3-Del-PR	ATGACGTCCAGGGCGTCCGTCGTCGACTTCGGCGCCGCG tgtaggctggagctgcttc	For disrupting *acnU3*
acnU3-Ts-PF	TAAGCCCGTTCGCTCTCCCG	For verifying mutant Δ*acnU3*

acnU3-Ts-PR	TCCAGGGCGTCCGTCGTCGA	
acnU4-Del-PF	TCATGACGTACCCGTCAGATCGACGACACGATCGACGGC attccggggatccgtcgacc	
acnU4-Del-PR	ATGGACGACGCCGCTTTCTCACAACTGCTGCTGAGCGAG tgtaggctggagctgcttc	For disrupting *acnU4*
acnU4-Ts-PF	TGACGTACCCGTCAGATCGAC	For verifying mutant Δ*acnU4*
acnU4-Ts-PR	ACGCCGCTTTCTCACAACT	
acnD–E-Del-PF	TACGAGGCTCAGCGGGCGCATGTCGGTCCAGTGCTCCTC attccggggatccgtcgacc	For disrupting *acnD-E*
acnD–E-Del-PR	ACATCAGGGCGATTCTCTGCGAGGGCGCGGGACTCGGAC tgtaggctggagctgcttc	
acnD–E-Ts-PF	TCAGCGGGCGCATGTCGGTC	For verifying mutant Δ*acnD-E*
acnD–E-Ts-PR	ATTCTCTGCGAGGGCGCGGG	
acnN1-Del-PF	GGTCACCCGGGGGTGCTCCAGCAGAACGGTCTCCACCTC attccggggatccgtcgacc	
acnN1-Del-PR	GTGGTTCTGGTCGACTTCCGGCTGAAGCCGGCCGAATAC tgtaggctggagctgcttc	For disrupting *acnN1*
acnN1-Ts-PF	TGCTCCAGCAGAACGGTCTC	For verifying mutant Δ*acnN1*
acnN1-Ts-PR	ACTTCCGGCTGAAGCCGGC	
acnN3-Del-PF	GCCACTCCGCAGGAACAGGTCGTGTGCGAGCTGTTCGCG attccggggatccgtcgacc	
acnN3-Del-PR	TCACTCGTCGGCGGCGGCGGTGAGTTCGTGGAGCTTGGC tgtaggctggagctgcttc	For disrupting *acnN3*
acnN3-Ts-PF	ACTCCGCAGGAACAGGTCGT	For verifying mutant Δ*acnN3*
acnN3-Ts-PR	TGAGTTCGTGGAGCTTGGC	
acnF-Del-PF	ATGCTGACCGACGTGCTCTCCGTCGAGCTGGCCACGGCC attccggggatccgtcgacc	For disrupting *acnF*
acnF-Del-PR	TCACTCGGCCTCCGGCCGGTGCGCGGCGCATGCCTTCTC tgtaggctggagctgcttc	
acnF-Ts-PF	ACCGACGTGCTCTCCGTCGAG	For verifying mutant Δ*acnF*
acnF-Ts-PR	GCGCGGCGCATGCCTTCTC	
acnG–P-Del-PF	ATGCGGCGCACCGCGGCCCGGGAGCGCGCGCTGACCGGC attccggggatccgtcgacc	
acnG–P-Del-PR	CGAGACCAGCACGAGTTGGATCGCGTCGCCACGGGCGAT tgtaggctggagctgcttc	For disrupting *acnG-P*
acnG–P-Ts-PF	ACCGCGGCCCGGGAGCGCGC	For verifying mutant Δ*acnG-P*

acnG–P-Ts-PR	AGTTGGATCGCGTCGCCACG	
acnR-Del-PF	CCGAAAGGCTTCCTCGGCCTCCCGCTCCACGGGCGGCGCACTAGTattccggggatccgtcgacc	For disrupting *acnR*
acnR-Del-PR	GGCATCACAGTCGCCGAGGAGCAGGGAATGGCGGCCCTGACTAGTtgtaggctggagctgcttc	For disrupting *acnR*
acnR-Ts-PF	GTACCAATGATCGGTGGCTG	For verifying mutant Δ*acnR*
acnR-Ts-PR	CTTACGCTGTAAGGAGATGCTC	
acnQ-Del-PF	GACCAGCAGGTCAAGCTCTGCTTCCCCCGGCTCGGCCAGACTAGTattccggggatccgtcgacc	
acnQ-Del-PR	CAGCTTGAACCGCCAGTACCCGCTGAACTCGATGGACCGACTAGTtgtaggctggagctgcttc	For disrupting *acnQ*
acnQ-Ts-PF	GATGCCCAGCACTCTTGGAAG	For verifying mutant Δ*acnQ*
acnQ-Ts-PR	CTTTCGTGCGTACCGCTTCTG	
acnT1-Del-PF	GCAGTTCACCCTTCGCGGGCGCACCGTCGACGCGGTGAAattccggggatccgtcgacc	
acnT1-Del-PR	GAGGGTGGGCCGGTTCACCTTGACCGCGGTCATGGTGATtgtaggctggagctgcttc	For disrupting *acnT1*
acnT1-Ts-PF	TTCACCCTTCGCGGGCGCAC	For verifying mutant Δ*acnT1*
acnT1-Ts-PR	TTCACCTTGACCGCGGTCAT	
acnT2-Del-PF	TGACACCTGGCTGATCTTCAGCAGGGACATGAAGCTGTGattccggggatccgtcgacc	
acnT2-Del-PR	ACCCGTCAGCAGCCCCGTCGAGCCCATGTCGCCCCGGAAtgtaggctggagctgcttc	For disrupting *acnT2*
acnT2-Ts-PF	TGGCTGATCTTCAGCAGGGA	For verifying mutant Δ*acnT2*
acnT2-Ts-PR	AGCCCCGTCGAGCCCATGTC	
acnT3-Del-PF	TCTTCCAGCGGTTCGTGCGCTCGGCCCCCTGCGCGGACTattccggggatccgtcgacc	
acnT3-Del-PR	TGAACAGCGAGGCACTGACACCGTTGGCCTTGGCGAACAtgtaggctggagctgcttc	For disrupting *orf(T3)*
acnT3-Ts-PF	ACACCGTTGGCCTTGGCGAA	For verifying mutant Δ*acnT3*
acnT3-Ts-PR	CGGTGATCTCGTACCGCACA	
orf(+1)-Del-PF	TTGCCGCCACCGCTCCCCGGTGATCTCGTACCGCACACCattccggggatccgtcgacc	
orf(+1)-Del-PR	TTTCACGGCCGATGACGCCGACCTGCTGATCGAACTGGAtgtaggctggagctgcttc	For disrupting *orf(+1)*
orf(+1)-Ts-PF	CGGTGATCTCGTACCGCACA	
orf(+1)-Ts-PR	ACGCCGACCTGCTGATCGAA	

orf(+2)-Del-PF	GTGGGGGAAGGCCAGTTGGTCACGATGGCCCTGGACGGC attccggggatccgtcgacc	
orf(+2)-Del-PR	TCAGGCGGGGCGACGCCAGACCATGCGGACGGCGAAGTT tgtaggctggagctgcttc	For disrupting *orf(+2)*
orf(+2)-Ts-PF	AAGGCCAGTTGGTCACGAT	
orf(+2)-Ts-PR	GACGCCAGACCATGCGGACG	
orf(+3)-Del-PF	TGTTTCCGCCGGCCGACTCCCTGCCGAGCCCCGACGTCA attccggggatccgtcgacc	
orf(+3)-Del-PR	TGAGCATTTCCTGGGTCCGCTGCTGCTGGAGTATCTCCG tgtaggctggagctgcttc	For disrupting *orf(+3)*
orf(+3)-Ts-PF	TTTCCGCCGGCCGACTCCCT	For verifying mutant Δ*orf(-3)*
orf(+3)-Ts-PR	CATTTCCTGGGTCCGCTGCT	
PHS-Del-PF	GTGGATGTGCATGGGGTGCACGATGGGCGCGAGGTTGAGAC TAGTattccggggatccgtcgacc	
PHS-Del-PR	GACCGCAATCTCGACACCGACGAGGACGGACGGCTCAACA CTAGTtgtaggctggagctgcttc	For disrupting *PHS*
PHS-Ts-PF	GGCAGTGGTACATGAACCTG	For verifying mutant Δ*PHS*
PHS-Ts-PR	CGAACTGCGGCCGTATGTC	

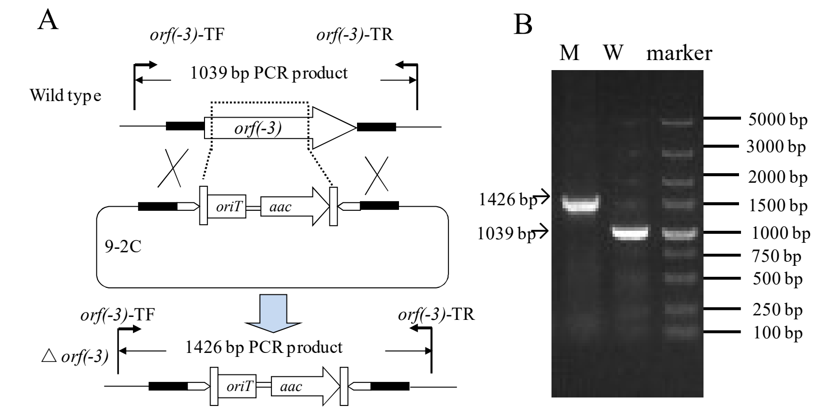

Figure S1. Disruption of *orf(-3)* in WT *S. costaricanus* SCSIO ZS0073 via PCR-targeting. (A) Schematic representation for disruption of *orf(-3)*. (B) PCR analyses of the WT strain and the *orf(-3)* double-cross mutant carried out using the primers listed in **Table S3**. Marker: DNA molecular ladder; W: using the genomic DNA of *S. costaricanus* SCSIO ZS0073 as template; M: using the genomic DNA of *orf(-3)* mutant as template.

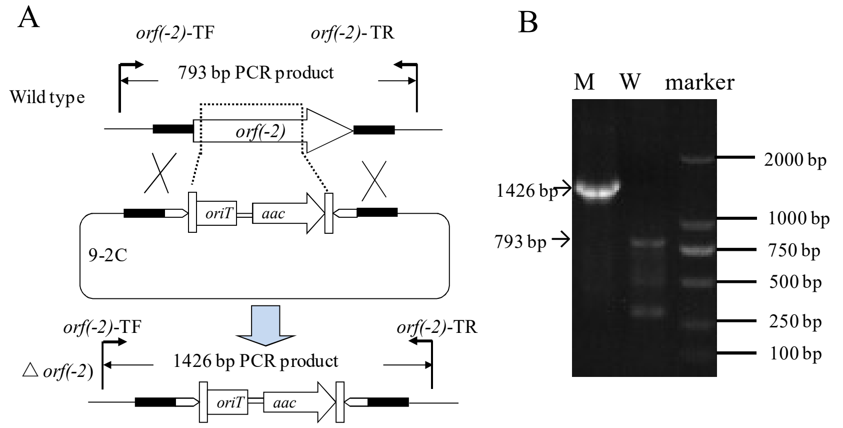

Figure S2. Disruption of *orf(-2)* in WT *S. costaricanus* SCSIO ZS0073 via PCR-targeting. (A) Schematic representation for disruption of *orf(-2)*. (B) PCR analyses of the WT strain and the *orf(-2)* double-cross mutant carried out using the primers listed in **Table S3**. Marker: DNA molecular ladder; W: using the genomic DNA of *S. costaricanus* SCSIO ZS0073 as template; M: using the genomic DNA of *orf(-2)* mutant as template.

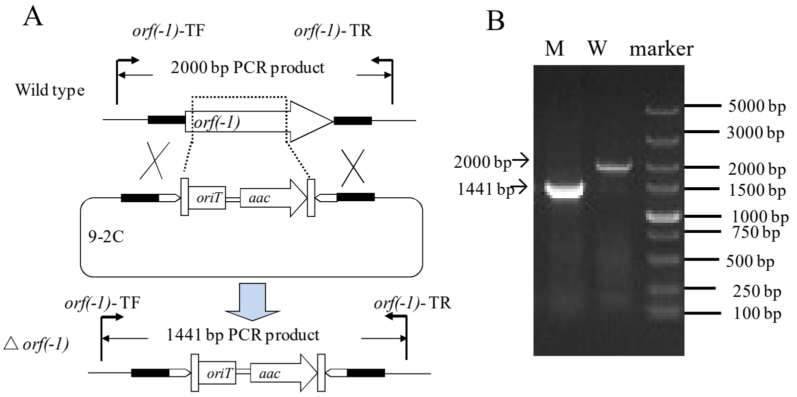

Figure S3. Disruption of *orf(-1)* in WT *S. costaricanus* SCSIO ZS0073 via PCR-targeting. (A) Schematic representation for disruption of *orf(-1)*. (B) PCR analyses of the WT strain and the *orf(-1)* double-cross mutant carried out using the primers listed in **Table S3**. Marker: DNA molecular ladder; W: using the genomic DNA of *S. costaricanus* SCSIO ZS0073 as template; M: using the genomic DNA of *orf(-1)* mutant as template.

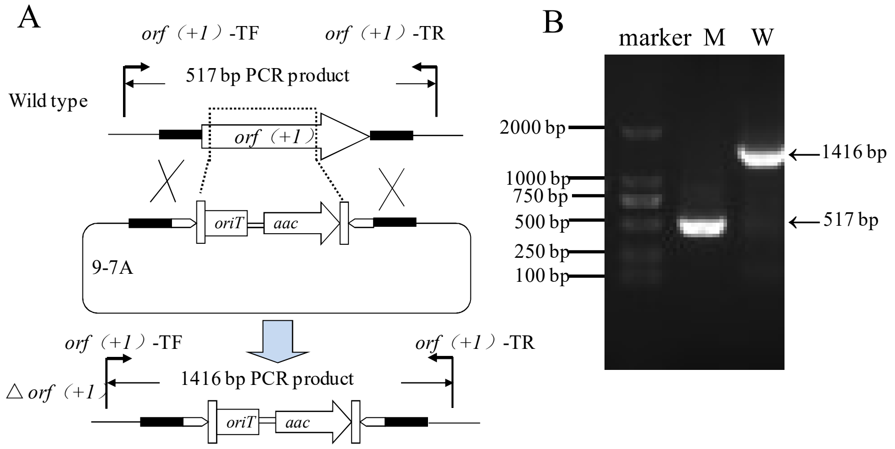

Figure S4. Disruption of *orf(+1)* in WT *S. costaricanus* SCSIO ZS0073 via PCR-targeting. (A) Schematic representation for disruption of *orf(+1)*. (B) PCR analyses of the WT strain and the *orf(+1)* double-cross mutant carried out using the primers listed in **Table S3**. Marker: DNA molecular ladder; W: using the genomic DNA of *S. costaricanus* SCSIO ZS0073 as template; M: using the genomic DNA of *orf(+1)* mutant as template.

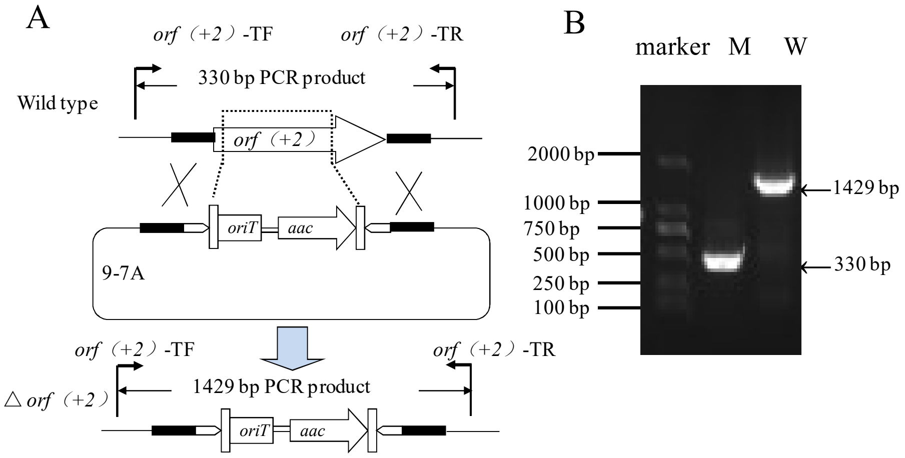

Figure S5. Disruption of *orf(+2)* in WT *S. costaricanus* SCSIO ZS0073 via PCR-targeting. (A) Schematic representation for disruption of *orf(+2)*. (B) PCR analyses of the WT strain and the *orf(+2)* double-cross mutant carried out using the primers listed in **Table S3**. Marker: DNA molecular ladder; W: using the genomic DNA of *S. costaricanus* SCSIO ZS0073 as template; M: using the genomic DNA of *orf(+2)* mutant as template.

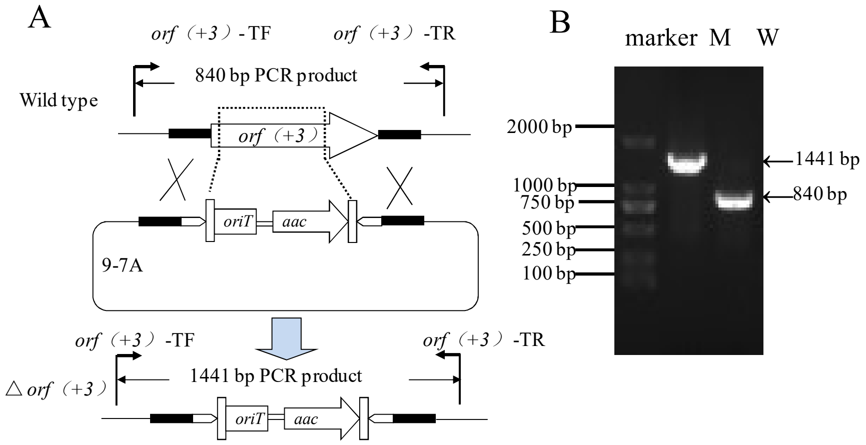

Figure S6. Disruption of *orf(+3)* in WT *S. costaricanus* SCSIO ZS0073 via PCR-targeting. (A) Schematic representation for disruption of *orf(+3)*. (B) PCR analyses of the WT strain and the *orf(+3)* double-cross mutant carried out using the primers listed in **Table S3**. Marker: DNA molecular ladder; W: using the genomic DNA of *S. costaricanus* SCSIO ZS0073 as template; M: using the genomic DNA of *orf(+3)* mutant as template.

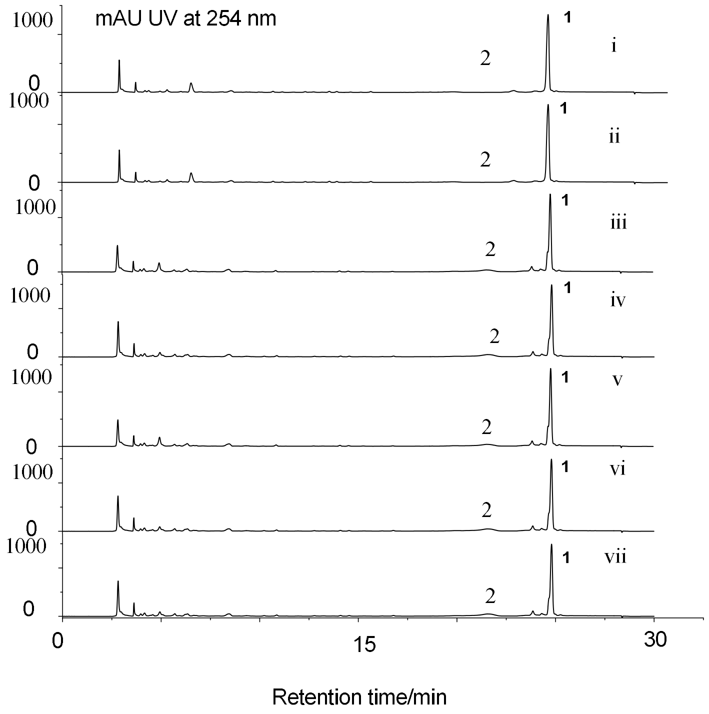

Figure S7. HPLC analyses of fermentation extracts. 1) WT, 2) Δ*orf*（*-3*）mutant, 3) Δ*orf*（*-2*）mutant, 4) Δ*orf*（*-1*）mutant, 5) Δ*orf*（*+1*）mutant, 6) Δ*orf*（*+2*）mutant, 7) Δ*orf*（*+3*）mutant . **1**: actinomycin D, **2**: actinomycin Xoβ

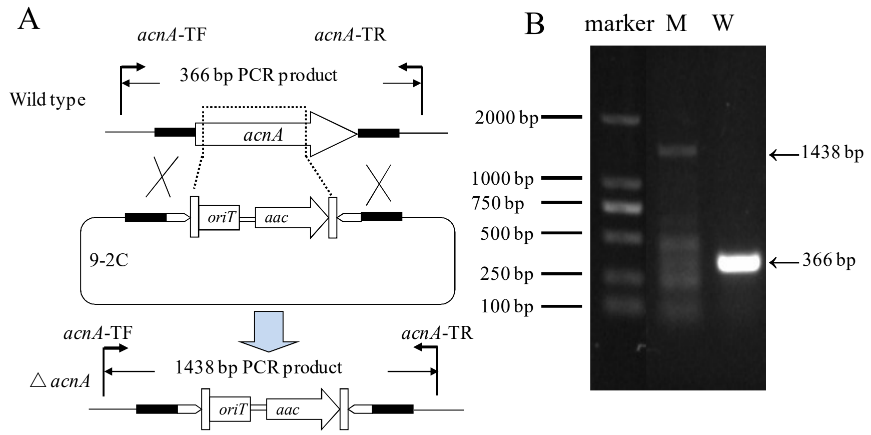

Figure S8. Disruption of *acnA* in WT *S. costaricanus* SCSIO ZS0073 via PCR-targeting. (A) Schematic representation for disruption of *acnA*. (B) PCR analyses of the WT strain and the *acnA* double-cross mutant carried out using the primers listed in **Table S3**. Marker: DNA molecular ladder; W: using the genomic DNA of *S. costaricanus* SCSIO ZS0073 as template; M: using the genomic DNA of *acnA* mutant as template.

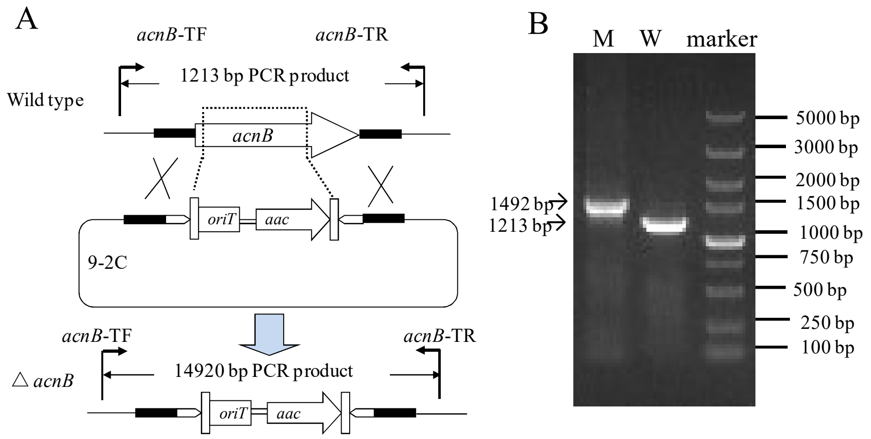

Figure S9. Disruption of *acnB* in WT *S. costaricanus* SCSIO ZS0073 via PCR-targeting. (A) Schematic representation for disruption of *acnB*. (B) PCR analyses of the WT strain and the *acnB* double-cross mutant carried out using the primers listed in **Table S3**. Marker: DNA molecular ladder; W: using the genomic DNA of *S. costaricanus* SCSIO ZS0073 as template; M: using the genomic DNA of *acnB* mutant as template.

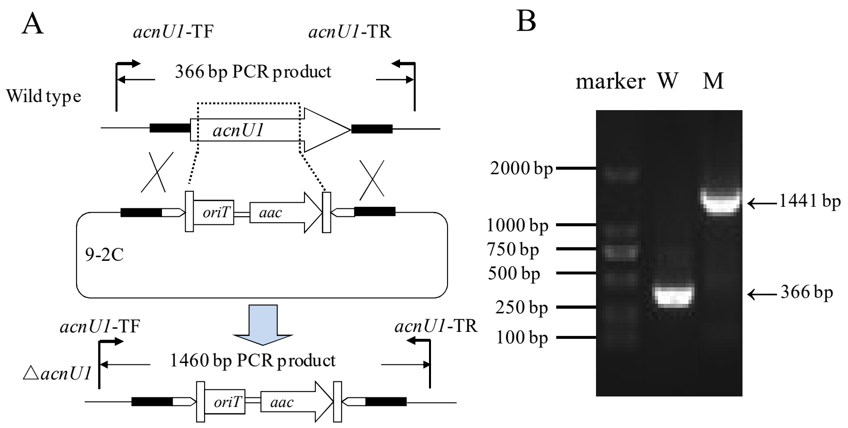

Figure S10. Disruption of *acnU1* in WT *S. costaricanus* SCSIO ZS0073 via PCR-targeting. (A) Schematic representation for disruption of *acnU1*. (B) PCR analyses of the WT strain and the *acnU1* double-cross mutant carried out using the primers listed in **Table S3**. Marker: DNA molecular ladder; W: using the genomic DNA of *S. costaricanus* SCSIO ZS0073 as template; M: using the genomic DNA of *acnU1* mutant as template.

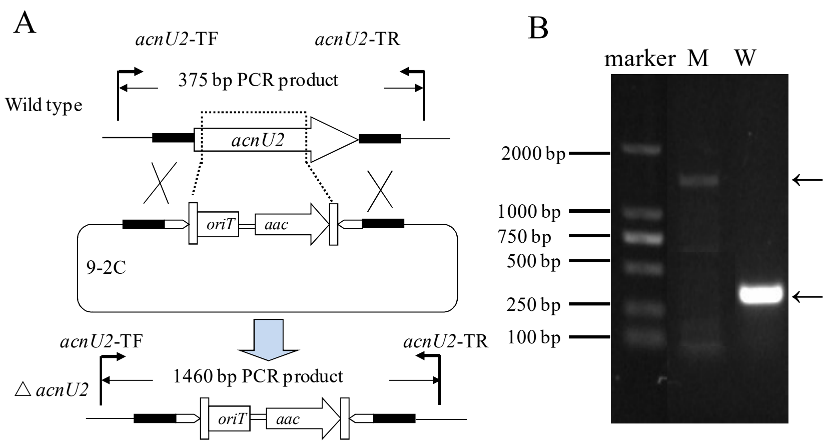

Figure S11. Disruption of *acnU2* in WT *S. costaricanus* SCSIO ZS0073 via PCR-targeting. (A) Schematic representation for disruption of *acnU2*. (B) PCR analyses of the WT strain and the *acnU2* double-cross mutant carried out using the primers listed in **Table S3**. Marker: DNA molecular ladder; W: using the genomic DNA of *S. costaricanus* SCSIO ZS0073 as template; M: using the genomic DNA of *acnU2* mutant as template.

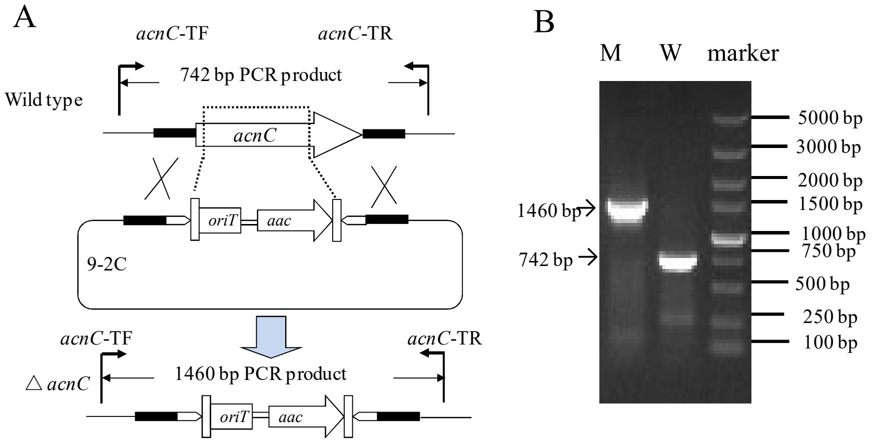

Figure S12. Disruption of acnC in WT S. costaricanus SCSIO ZS0073 via PCR-targeting. (A) Schematic representation for disruption of acnC. (B) PCR analyses of the WT strain and the acnC double-cross mutant carried out using the primers listed in **Table S3**. Marker: DNA molecular ladder; W: using the genomic DNA of *S. costaricanus* SCSIO ZS0073 as template; M: using the genomic DNA of *acnC* mutant as template.

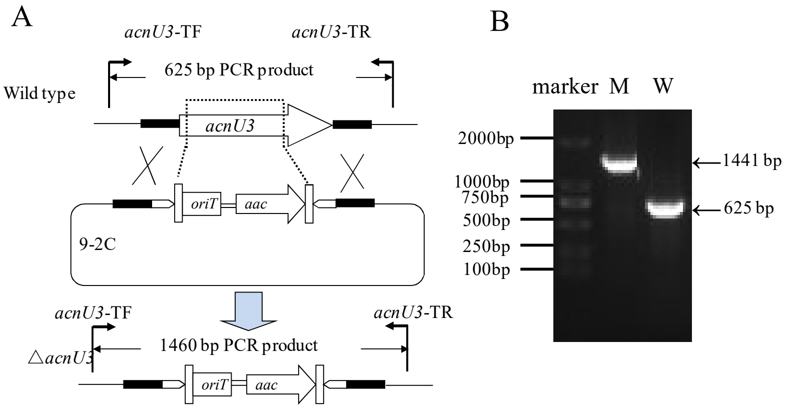

Figure S13. Disruption of *acnU3* in WT *S. costaricanus* SCSIO ZS0073 via PCR-targeting. (A) Schematic representation for disruption of *acnU3*. (B) PCR analyses of the WT strain and the *acnU3* double-cross mutant carried out using the primers listed in **Table S3**. Marker: DNA molecular ladder; W: using the genomic DNA of *S. costaricanus* SCSIO ZS0073 as template; M: using the genomic DNA of *acnU3* mutant as template.

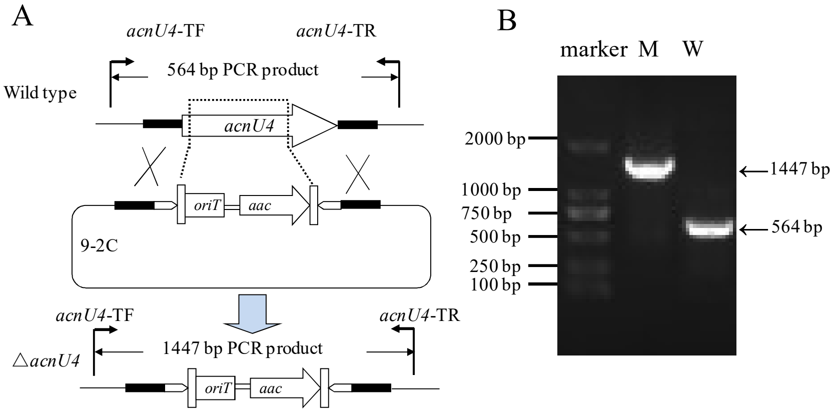

Figure S14. Disruption of *acnU4* in WT *S. costaricanus* SCSIO ZS0073 via PCR-targeting. (A) Schematic representation for disruption of *acnU4*. (B) PCR analyses of the WT strain and the *acnU4* double-cross mutant carried out using the primers listed in **Table S3**. Marker: DNA molecular ladder; W: using the genomic DNA of *S. costaricanus* SCSIO ZS0073 as template; M: using the genomic DNA of *acnU4* mutant as template.

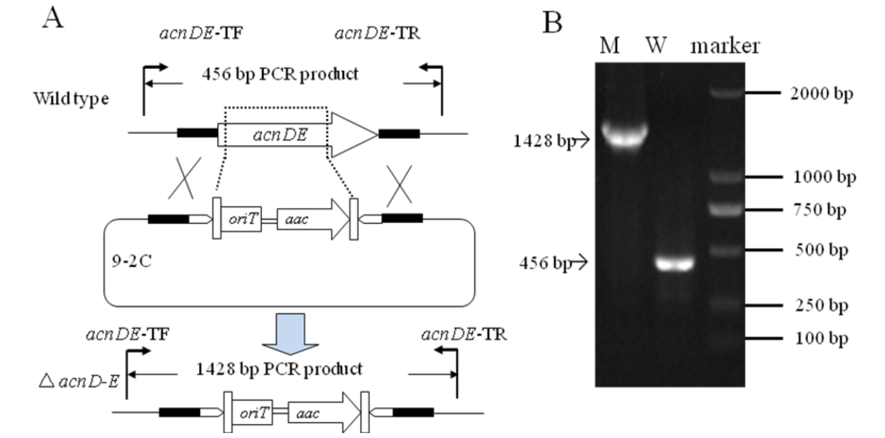

Figure S15. Disruption of *acnD–E* in WT *S. costaricanus* SCSIO ZS0073 via PCR-targeting. (A) Schematic representation for disruption of *acnD–E*. (B) PCR analyses of the WT strain and the *acnD–E* double-cross mutant carried out using the primers listed in **Table S3**. Marker: DNA molecular ladder; W: using the genomic DNA of *S. costaricanus* SCSIO ZS0073 as template; M: using the genomic DNA of *acnD–E* mutant as template.

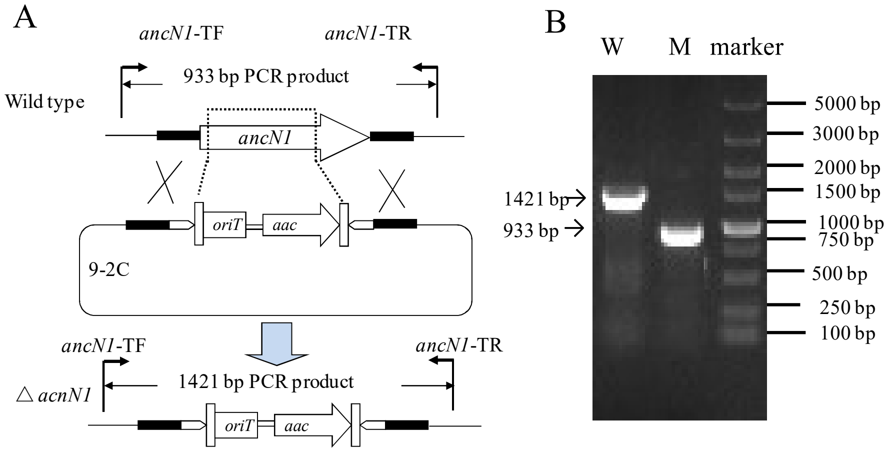

Figure S16. Disruption of *acnN1* in WT *S. costaricanus* SCSIO ZS0073 via PCR-targeting. (A) Schematic representation for disruption of *acnN1*. (B) PCR analyses of the WT strain and the *acnN1* double-cross mutant carried out using the primers listed in **Table S3**. Marker: DNA molecular ladder; W: using the genomic DNA of *S. costaricanus* SCSIO ZS0073 as template; M: using the genomic DNA of *acnN1* mutant as template.

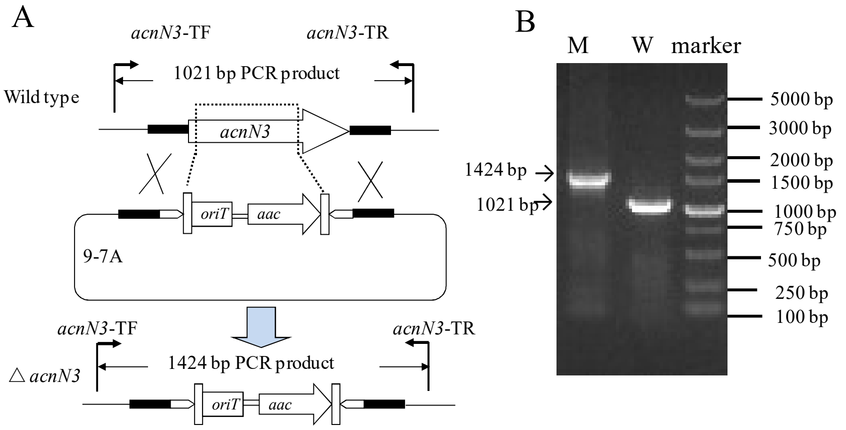

Figure S17. Disruption of *acnN3* in WT *S. costaricanus* SCSIO ZS0073 via PCR-targeting. (A) Schematic representation for disruption of *acnN3*. (B) PCR analyses of the WT strain and the *acnN3* double-cross mutant carried out using the primers listed in **Table S3**. Marker: DNA molecular ladder; W: using the genomic DNA of *S. costaricanus* SCSIO ZS0073 as template; M: using the genomic DNA of *acnN3* mutant as template.

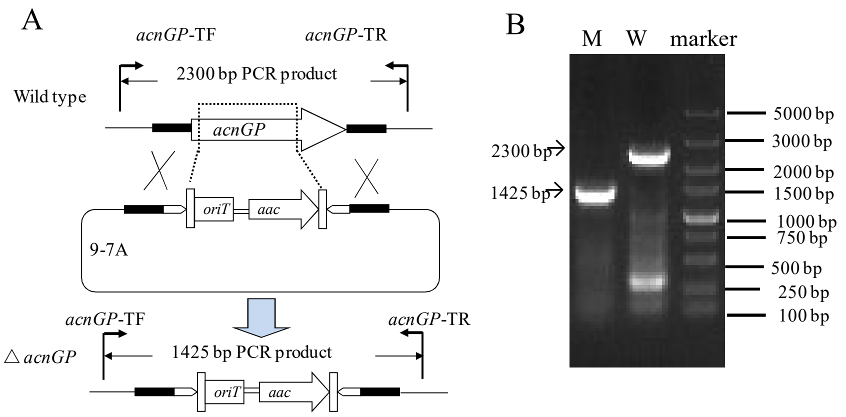

Figure S18. Disruption of *acnG–P* in WT *S. costaricanus* SCSIO ZS0073 via PCR-targeting. (A) Schematic representation for disruption of *acnG–P*. (B) PCR analyses of the WT strain and the *acnG–P* double-cross mutant carried out using the primers listed in **Table S3**. Marker: DNA molecular ladder; W: using the genomic DNA of *S. costaricanus* SCSIO ZS0073 as template; M: using the genomic DNA of *acnG–P* mutant as template.

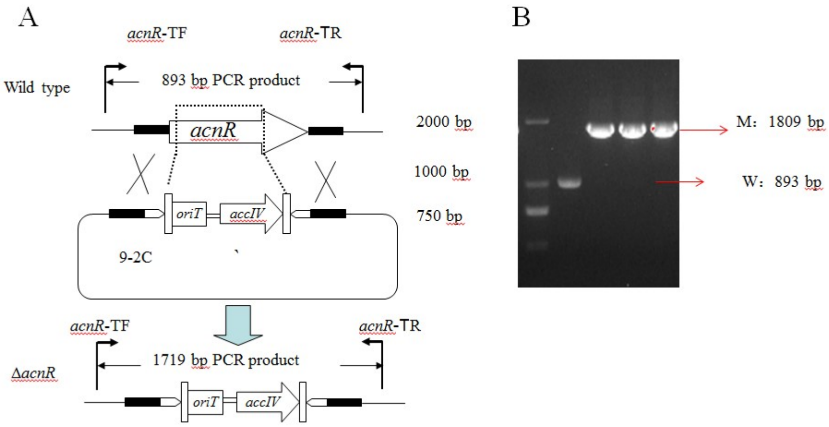

Figure S19. Disruption of *acnR* in WT *S. costaricanus* SCSIO ZS0073 via PCR-targeting. (A) Schematic representation for disruption of *acnR*. (B) PCR analyses of the WT strain and the *acnR* double-cross mutant carried out using the primers listed in **Table S3**. Marker: DNA molecular ladder; W: using the genomic DNA of *S. costaricanus* SCSIO ZS0073 as template; M: using the genomic DNA of *acnR* mutant as template.

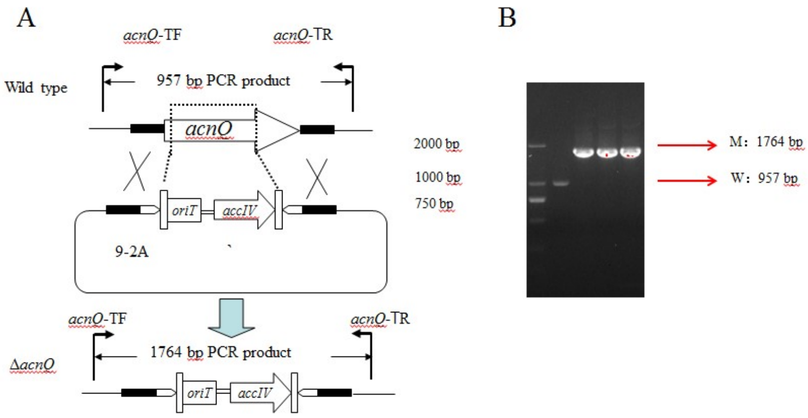

Figure S20. Disruption of *acnQ* in WT *S. costaricanus* SCSIO ZS0073 via PCR-targeting. (A) Schematic representation for disruption of *acnQ*. (B) PCR analyses of the WT strain and the *acnQ* double-cross mutant carried out using the primers listed in **Table S3**. Marker: DNA molecular ladder; W: using the genomic DNA of *S. costaricanus* SCSIO ZS0073 as template; M: using the genomic DNA of *acnQ* mutant as template.

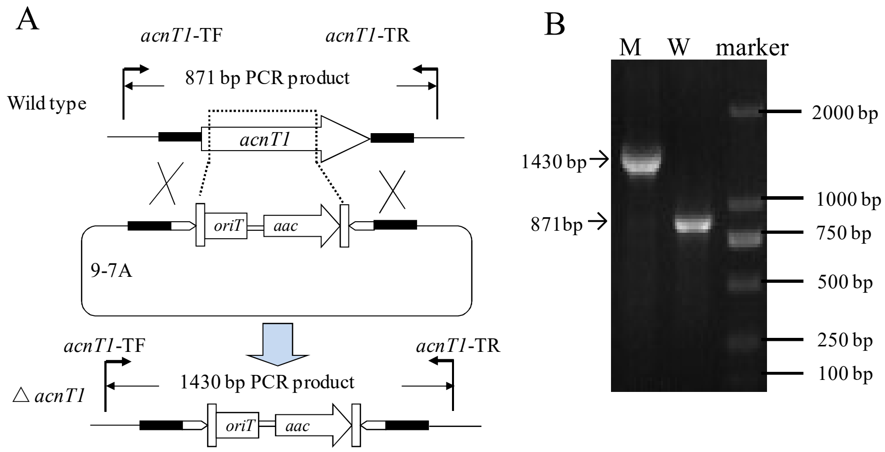

Figure S21. Disruption of *acnT1* in WT *S. costaricanus* SCSIO ZS0073 via PCR-targeting. (A) Schematic representation for disruption of *acnT1*. (B) PCR analyses of the WT strain and the *acnT1* double-cross mutant carried out using the primers listed in **Table S3**. Marker: DNA molecular ladder; W: using the genomic DNA of *S. costaricanus* SCSIO ZS0073 as template; M: using the genomic DNA of *acnT1* mutant as template.

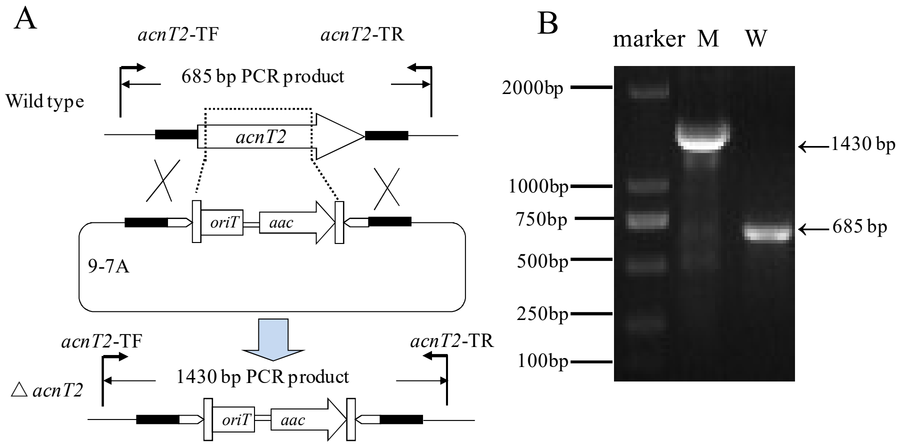

Figure S22. Disruption of *acnT2* in WT *S. costaricanus* SCSIO ZS0073 via PCR-targeting. (A) Schematic representation for disruption of *acnT2*. (B) PCR analyses of the WT strain and the *acnT2* double-cross mutant carried out using the primers listed in **Table S3**. Marker: DNA molecular ladder; W: using the genomic DNA of *S. costaricanus* SCSIO ZS0073 as template; M: using the genomic DNA of *acnT2* mutant as template.

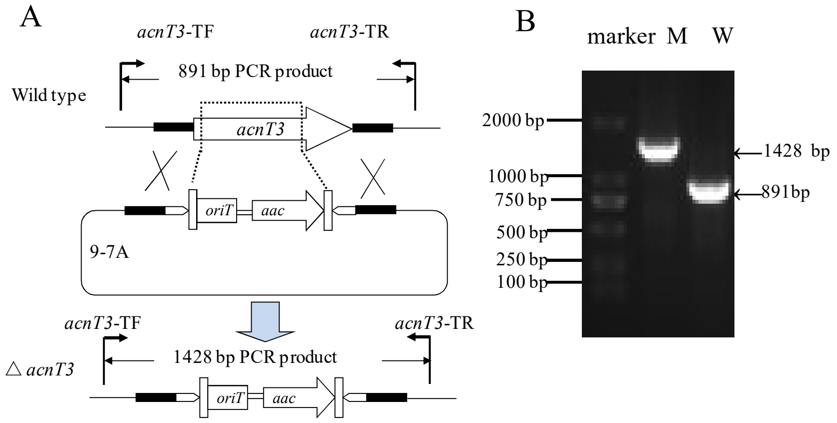

Figure S23. Disruption of *acnT3* in WT *S. costaricanus* SCSIO ZS0073 via PCR-targeting. (A) Schematic representation for disruption of *acnT3*. (B) PCR analyses of the WT strain and the *acnT3* double-cross mutant carried out using the primers listed in **Table S3**. Marker: DNA molecular ladder; W: using the genomic DNA of *S. costaricanus* SCSIO ZS0073 as template; M: using the genomic DNA of *acnT3* mutant as template.

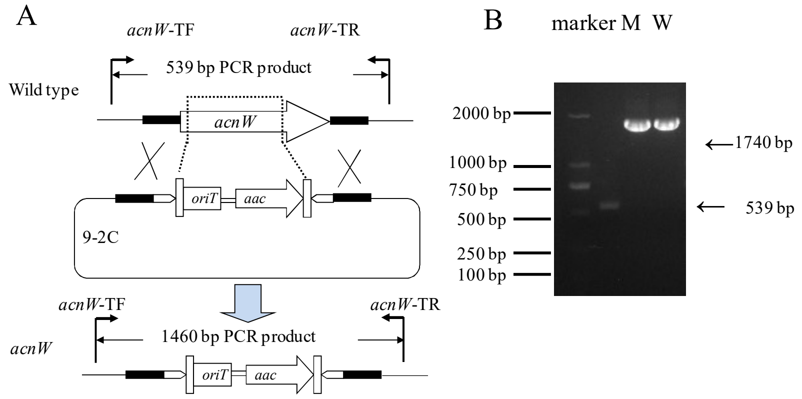

Figure S24. Disruption of *acnW* in WT *S. costaricanus* SCSIO ZS0073 via PCR-targeting. (A) Schematic representation for disruption of *acnW*. (B) PCR analyses of the WT strain and the *acnW* double-cross mutant carried out using the primers listed in **Table S3**. Marker: DNA molecular ladder; W: using the genomic DNA of *S. costaricanus* SCSIO ZS0073 as template; M: using the genomic DNA of *acnW* mutant as template.

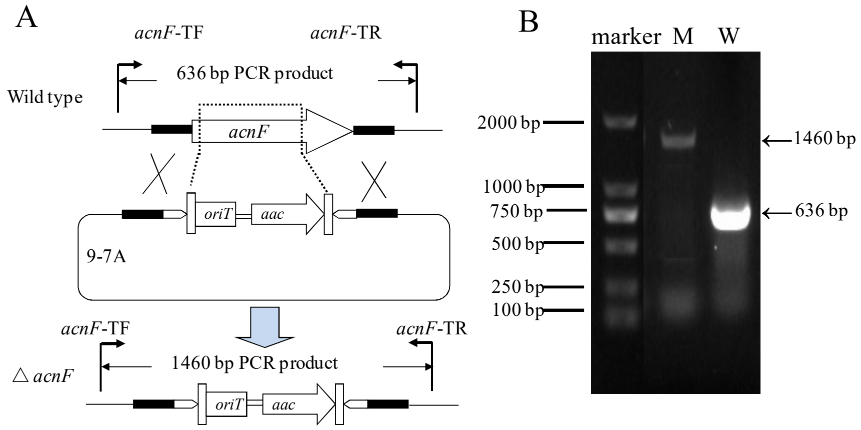

Figure S25. Disruption of *acnF* in WT *S. costaricanus* SCSIO ZS0073 via PCR-targeting. (A) Schematic representation for disruption of *acnF*. (B) PCR analyses of the WT strain and the *acnF* double-cross mutant carried out using the primers listed in **Table S3**. Marker: DNA molecular ladder; W: using the genomic DNA of *S. costaricanus* SCSIO ZS0073 as template; M: using the genomic DNA of *acnF* mutant as template.

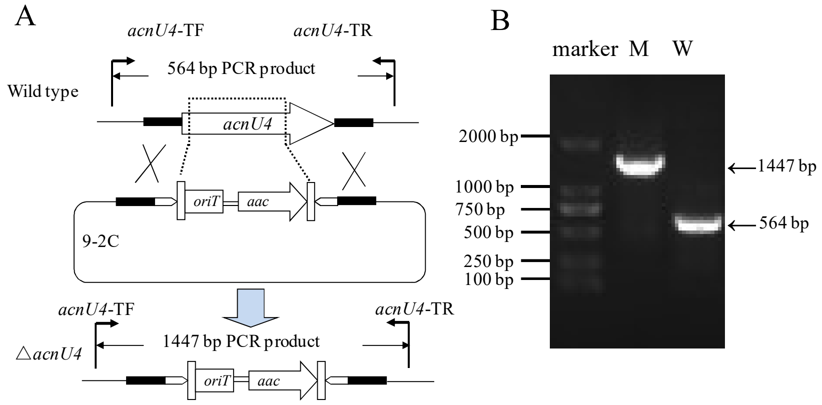

Figure S26. Disruption of *acnU4* in WT *S. costaricanus* SCSIO ZS0073 via PCR-targeting. (A) Schematic representation for disruption of *acnU4*. (B) PCR analyses of the WT strain and the *acnU4* double-cross mutant carried out using the primers listed in **Table S3**. Marker: DNA molecular ladder; W: using the genomic DNA of *S. costaricanus* SCSIO ZS0073 as template; M: using the genomic DNA of *acnU4* mutant as template.

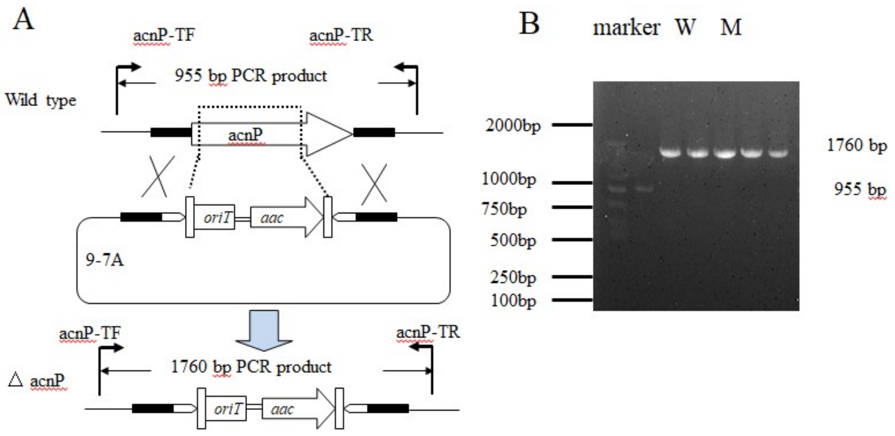

Figure S27. Disruption of *acnP* in WT *S. costaricanus* SCSIO ZS0073 via PCR-targeting. (A) Schematic representation for disruption of *acnP*. (B) PCR analyses of the WT strain and the *acnP* double-cross mutant carried out using the primers listed in **Table S3**. Marker: DNA molecular ladder; W: using the genomic DNA of *S. costaricanus* SCSIO ZS0073 as template; M: using the genomic DNA of *acnP* mutant as template.

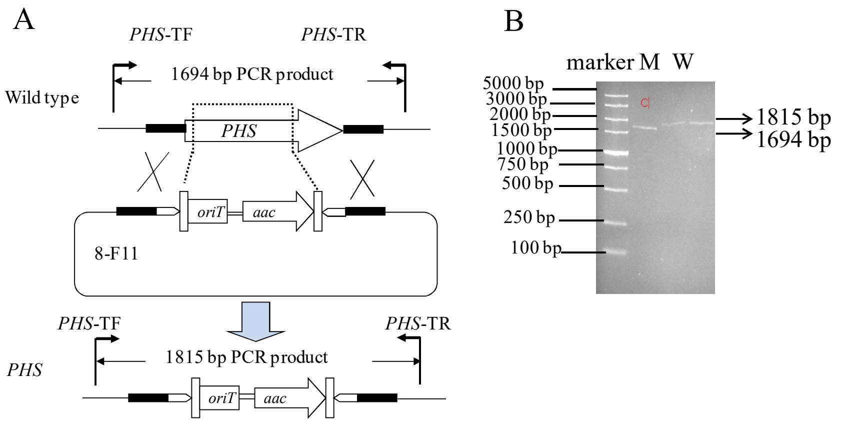

Figure S28. Disruption of *phs* in WT *S. costaricanus* SCSIO ZS0073 via PCR-targeting. (A) Schematic representation for disruption of *phs*. (B) PCR analyses of the WT strain and the *phs* double-cross mutant carried out using the primers listed in **Table S3**. Marker: DNA molecular ladder; W: using the genomic DNA of *S. costaricanus* SCSIO ZS0073 as template; M: using the genomic DNA of *phs* mutant as template.

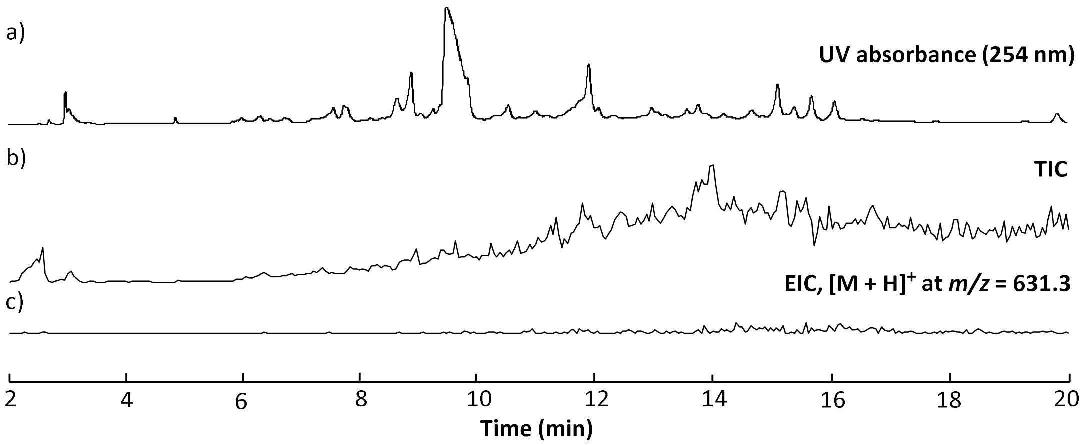

Figure S29. HPLC-ESI-MS chromatogram of the fermentation extract of Δ*acnF*. a) HPLC profile of the extract of Δ*acnF* (λ = 254 nm); b) total ion chromatogram (TIC) of the extract of Δ*acnF*; c) extract ion chromatogram (EIC) of the pentapeptide lactone monomer with $[M + H]^+$ at *m/z* 631.3.
